# The Role of Nomogram Based on the Combination of Ultrasound Parameters and Clinical Indicators in the Degree of Pathological Remission of Breast Cancer

**DOI:** 10.1155/2023/3077180

**Published:** 2023-02-16

**Authors:** Huangjing Chen, Hongyan Qian, Guifang Chen, Pengfei Zhu, Chunjuan Sun, Xiaotian Wu, Ying He

**Affiliations:** ^1^Medical College of Nantong University, Nantong 226361, Jiangsu, China; ^2^Key Laboratory of Cancer Research Center Nantong, Tumor Hospital Affiliated to Nantong University, Nantong 226006, Jiangsu, China; ^3^Department of Ultrasound, Tumor Hospital Affiliated to Nantong University, Nantong 226006, Jiangsu, China

## Abstract

**Background:**

The mortality rate of breast cancer (BC) ranks first among female tumors worldwide and presents a trend of younger age, which poses a great threat to women's health and life. Neoadjuvant chemotherapy (NAC) for breast cancer is defined as the first step of treatment for breast cancer patients without distant metastasis before planned surgical treatment or local treatment with surgery and radiotherapy. According to the current NCCN guidelines, patients with different molecular types of BC should receive neoadjuvant chemotherapy (NAC), which can not only achieve tumor downstaging, increase the chance of surgery, and improve the breast-conserving rate. In addition, it can identify new genetic pathways and drugs related to cancer, improve patient survival rate, and make new progress in breast cancer management.

**Objective:**

To explore the role of the nomogram established by the combination of ultrasound parameters and clinical indicators in the degree of pathological remission of breast cancer.

**Methods:**

A total of 147 breast cancer patients who received neoadjuvant chemotherapy and elective surgery in the Department of Ultrasound, Nantong Cancer Hospital, from May 2014 to August 2021 were retrospectively included. Postoperative pathological remission was divided into two groups according to Miller–Payne classification: no significant remission group (NMHR group, *n* = 93) and significant remission group (MHR group, *n* = 54). Clinical characteristics of patients were recorded and collected. The multivariate logistic regression model was used to screen the information features related to the MHR group, and then, a nomogram model was constructed; ROC curve area, consistency index (C-index, CI), calibration curve, and H-L test were used to evaluate the model. And the decision curve is used to compare the net income of the single model and composite model.

**Results:**

Among 147 breast cancer patients, 54 (36.7%) had pathological remission. Multivariate logistic regression showed that ER, reduction/disappearance of strong echo halo, Adler classification after NAC, PR + CR, and morphological changes were independent risk factors for pathological remission (*P* < 0.05). Based on these factors, the nomogram was constructed and verified. The area under the curve (AUC) and CI were 0.966, the sensitivity and specificity were 96.15% and 92.31%, and the positive predictive value (PPV) and negative predictive value (NPV) were 87.72% and 97.15%, respectively. The mean absolute error of the agreement between the predicted value and the real value is 0.026, and the predicted risk is close to the actual risk. In the range of HRT of about 0.0∼0.9, the net benefit of the composite evaluation model is higher than that of the single model. H-L test results showed that *χ*^2^ = 8.430, *P*=0.393 > 0.05.

**Conclusion:**

The nomogram model established by combining the changes of ultrasound parameters and clinical indicators is a practical and convenient prediction model, which has a certain value in predicting the degree of pathological remission after neoadjuvant chemotherapy.

## 1. Introduction

Breast cancer (BC) has the highest mortality rate among female tumors worldwide, with an incidence rate of about 23%, posing a great threat to women's health and life [1, 2]. Neoadjuvant chemotherapy (NAC) is an important part of comprehensive therapy for BC patients with different molecular types, which can not only achieve tumor downstaging, increase the chance of surgery, and improve the breast-conserving rate. In addition, new genetic pathways and drugs related to cancer can be identified to improve the survival rate of patients and make new progress in BC management [3–7]. At present, the WHO and Response Evaluation Criteria In Solid Tumors (RECIST) are the most tumors used in clinical evaluation of NAC tumors [8–9], and all of them have their own advantages. The gold standard for NAC response has always been pathological assessment [10], including residual cancer burden (RCB) [11] and Miller-Payne score system [12]. Relevant data show that the pathological complete remission rate is 3% to 30% [13], not all patients are sensitive to NAC, and some patients may gradually develop drug resistance during chemotherapy, which limits the clinical efficacy of drugs and leads to treatment failure. Some patients (less than 5%) may progress during neoadjuvant therapy and even lose the opportunity to receive surgery [14]. Therefore, it is more and more important to accurately monitor and evaluate the efficacy of NAC and to observe the sensitivity of patients with advanced breast cancer to chemotherapy drugs, so as to realize individualized treatment and improve the breast-conserving rate and survival period of patients [15]. Imaging examination can not only evaluate the efficacy, pathological status, and prognosis of NAC but also help to select the most appropriate surgical method. Multiple imaging evaluation methods, including magnetic resonance imaging (MRI), computed tomography (CT), positron emission tomography (PET-CT), mammography (MM), and ultrasound (US), have been widely used around the world. Current studies generally believe that MRI is more objective than the US, and its characteristics of tomography make the lesion display more accurately and have advantages in predicting the degree of pathological remission and prognosis of the primary lesion. However, due to the high cost, it has not been fully popularized around the world, especially in developing countries [16–19]. At the same time, some studies have shown that breast cancer patients with ER negative before NAC and high expression of Ki67 are more sensitive to chemotherapy and benefit more after chemotherapy, which may be sensitive factors to predict the efficacy of chemotherapy [20, 21]. Studies have shown that many changes will occur in ultrasound images of patients with pcR after NAC, such as PR and CR in clinical efficacy evaluation, attenuation and disappearance of posterior echo, elevation of internal echo, narrowing or disappearance of strong echo halo around the tumor, all of which are effective related indicators of tumor NAC [22]. In order to develop a clinical applicable, cost-effective, and easy to promote the new approach, this study will be commonly used two-dimensional gray-scale ultrasound, color Doppler flow imaging (CDFI), and clinical common testing of immunohistochemical and serum index together, to develop and validate based on ultrasonic features and clinical pathology nomogram, and to predict the postoperative pathological remission after NAC. Based on the changes of various parameters in the MHR group after the end of NAC, we believe that the changes in these ultrasound parameters most intuitively reflect the changes of tumors during the entire process of NAC and are closely related to the degree of pathological remission. The aim of this study was to explore the role of the nomogram established by the combination of ultrasound parameters and clinical indicators in the degree of pathological remission of breast cancer.

## 2. Materials and Methods

### 2.1. Basic Information

**Figure 1:**
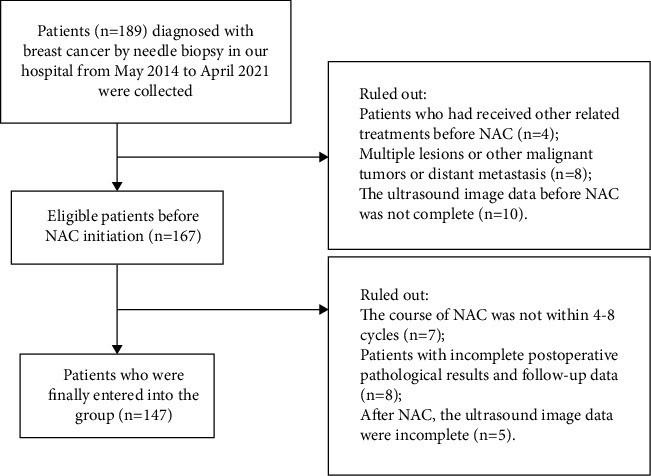
Flowchart of the study.

This study retrospectively collected 147 breast cancer patients who underwent neoadjuvant chemotherapy and elective surgery in the Ultrasound Department of Nantong Cancer Hospital from May 2014 to August 2021. Inclusion criteria were as follows: ① female; ② primary breast cancer, confirmed by ultrasound biopsy and in line with the diagnostic criteria of “Chinese Society of Clinical Oncology Breast Cancer Guideline version 2021: updates and interpretations” [23]; ③ TNM stage II to III, no disease distant metastases to contralateral or other organs. Exclusion criteria were as follows: ① patients who have received other related treatments before neoadjuvant chemotherapy; ② patients with multiple lesions and malignant tumors of other organs; ③ patients with incomplete clinical and imaging data; ④ the chemotherapy cycle is not within 4–8 cycles (Figure 1). This study has passed the ethics approval of the Medical Ethics Committee of Nantong Cancer Hospital, and patients' informed consent forms are exempted due to the retrospective nature of this study.

### 2.2. Methods

#### 2.2.1. Data Collection

Demographic and clinical data of patients were collected, including age, gender, menopause, history of childbearing and breastfeeding, family history of breast cancer (referring to immediate family members, including mothers, daughters, and sisters who have breast cancer), lymph node metastasis, and NAC course of treatment, NAC treatment plan, and breast cancer pathological type and stage.

#### 2.2.2. Ultrasound Image Acquisition and Evaluation

Before and after neoadjuvant chemotherapy, 2D gray-scale ultrasound and color Doppler examination were performed. Ultrasound-related parameters were collected, including diameter, morphology, aspect ratio, hyperechoic halo, calcification, tumor boundary, internal echo of the mass, posterior echo, Adler grade, and RI resistance index before and after NAC treatment.

Diameter is defined as the maximum diameter line. According to the efficacy evaluation criteria for solid tumors, the efficacy was divided into four parts by the change of focal diameter; complete response (CR) : all target lesions disappear and no new lesions appear; partial response (PR): the total baseline longest diameter of all target lesions was reduced by ≥30%; progression disease (PD): the total baseline maximum diameter of all target lesions increased by ≥20%; and stable disease (SD): the total length of baseline diameter of all target lesions decreased but did not reach PR or increased but did not reach PD. CR and PR were considered to have significant therapeutic effects, while SD and PD were not.

Morphological change observation whether the morphology becomes regular was defined as the shape of the mass becomes more regular after the end of NAC, which can be described by geometric shapes, with fewer lobulations and no angular protrusions.

Aspect ratio: an aspect ratio <1 indicates that the long axis of the lump is parallel to the skin and an aspect ratio >1 indicates that the front and rear diameter is greater than the horizontal diameter. The aspect ratio changes the anterior and posterior diameter and horizontal meridian of the mass change.

Calcification changes refer to the increase in the number of strong echo spots in the mass on two-dimensional gray-scale ultrasound images compared with that before NAC.

Tumor boundary changes refer to the fuzzy, angular, minute lobulation, and burr of the original sharp and clear tumor edges after the end of NAC.

Internal echogenicity: compared with the glandular tissue of the breast to determine the echogenicity of the mass, it can be classified as very low, low, mixed, or isoechoic. Echogenicity refers to the increased echogenicity of the mass compared to that before the onset of NAC.

Posterior echo attenuation is defined as the contrast between the echo in the depth of the tumor and the tissue echo at the same depth in the area around the tumor on the same section, which is lower than the tissue echo at the same depth in the surrounding area. It is generally believed that posterior echo attenuation can represent malignant signs. Similar ones are said to have no change in rear echo. The change of the posterior echo was located as an enhancement of the posterior echo compared to the ultrasound image before the NAC began.

Adler grading observes the distribution and richness of blood flow, finds the section with the most abundant blood flow, calculates the number of blood vessels, and defines the blood flow characteristics according to the semiquantitative grading of Adler: Level 0: no blood flow in the lesion; Level I: a small amount of blood flow, with 1 or 2 punctured or thin rod blood flow; Grade II: moderate blood flow, one major blood vessel can be seen, its length is close to or beyond the radius of the lesion or 3∼4 punctured or fine rod-shaped blood vessels; Grade III: abundant blood flow, visible more than 4 blood vessels or interconnected, intertwined into a network. The definition of Alder grading change is whether grading decreases after NAC.

Resistance index (RI) is the ratio of the difference between peak systolic and end diastolic velocity to peak systolic velocity, reflecting the distal resistance index of the vessel. RI was measured before and after NAC treatment to determine whether RI decreased.

#### 2.2.3. Collection and Evaluation of Immunohistochemical and Serum Indicators

The biopsy specimen of the breast mass was fixed and sent to the pathology department for immunohistochemistry. The results of immunohistochemical staining were evaluated by two senior pathologists, respectively, in a double-blind method with reference to the staining evaluation criteria proposed by Fromowitz et al. The percentage of positive cells in tumor cells was calculated to evaluate the status of ER (estrogen receptor), PR (progesterone receptor), proliferative nuclear antigen KI67, and human epidermal growth factor receptor C-erbb-2. ER, PR ≥1% was defined as ER, PR positive; Her-2 (0∼+) was determined as negative, her-2 (3+) was determined as positive, and HER-2(2+) was determined by fluorescence in situ hybridization. K-67 ≥ 20% was highly expressed.

After the first admission, 3 ml fasting venous blood was collected from the subjects in the morning before NAC, and the levels of CEA (carcinoembryonic antigen), sugar antigen CA153, sugar antigen CA125, and sugar antigen 50CA50 were determined by electrochemiluminescence immunoassay. After the treatment of NAC, the above indicators were tested again, and the results of the two tests were recorded.

### 2.3. Grouping and Evaluation

The outcome of this study was the degree of postoperative pathological remission after NAC. The degree of postoperative pathological remission was evaluated according to Miller–Payne's modified grading criteria for pathological response. Grade I: no change or slight change in tumor cells, no overall reduction, or no significant change; Grade II: the number of tumor cells decreased by <30%; Grade III: tumor cells reduced by 30%∼90%; Grade IV: reduction of tumor cells >90%, with only small clusters or widely dispersed residual cells; Grade V: no malignant cells in the tumor site, only fibrotic stroma. Grade I to III were nonmajor histological response (NMHR) groups, and grade IV to V were major histological response (MHR) groups.

### 2.4. Statistical Methods

SPSS 26.0 (Statistical Product and Service Solutions) and R Studio software were used for statistical analysis, Shapiro–Wilk test was used to test the normality of the data, and measurement data subject to normal distribution were expressed as mean ± standard deviation (mean ± SD). Measurement data that do not obey normal distribution are described by quartile M (P25, P75); enumeration data were described by [*n*(%)]; two independent samples *t*-test was used to compare the measurement data of the two groups with normal distribution, and Mann–Whitney *U* test was used to compare the measurement data of the two groups. The chi-square test was used for the comparison, and the variables with *P* < 0.05 in the univariate analysis results were used as independent variables in the multivariate analysis and were included in the multivariate logistic regression model analysis. The predicted probability of disease and the actual situation were plotted on the ROC curve, and the area under the curve was calculated. The independent risk factors were introduced into R Studio to establish a nomogram model for individualized prediction of disease, and the Bootstrap self-sampling method was used to conduct internal validation of the nomogram model. To measure the degree of discrimination of the model, the Hosmer–Lemeshow test is used to evaluate the model and the calibration curve to measure the degree of calibration of the model and used the decision curve to compare the net returns of the composite model and the single model.

## 3. Results

### 3.1. Baseline Data

From May 2014 to August 2021, a total of 189 candidates from Nantong Cancer Hospital were collected, and a total of 147 candidates met the inclusion criteria, including 93 in the NMHR group and 54 in the MHR group. The age of the NMHR group was greater than that of the MHR group (*P*=0.009). The rate of treatment ≥6 periods in the MHR group was higher than that in the NMHR group (*P*=0.015). The MHR group and NMHR group had different treatment regimens (*P* < 0.001). The positive rate of ER in the NMHR group was higher than that in the MHR group (*P* < 0.001). The positive rate of PR in the NMHR group was higher than that in the MHR group (*P* < 0.001). The positive rate of Ki67 was higher than that in the NMHR group (*P*=0.010) as shown in Table 1.

### 3.2. Comparative Analysis of Differences between Ultrasound and Serum Indexes before and after Treatment

Table 2 shows that the hyperechoic halo rate of the MHR group before treatment was higher than that of the NMHR group (*P* < 0.001); the posterior echo attenuation rate of the NMHR group before treatment was higher than that of the MHR group (*P*=0.013); MHR was displayed before and after treatment; Adler grades were different between the MHR group and NMHR group (both *P* < 0.001); RI and CA153 in the NMHR group were higher than those in the MHR group before treatment (*P* < 0.001 and *P*=0.003, respectively). After treatment, the hyperechoic halo rate of the NMHR group was higher than that of the MHR group (*P* < 0.001); the diameter, RI, CEA, and CA153 of the NMHR group were higher than those of the MHR group after treatment (*P* < 0.001, *P* < 0.001, *P*=0.003 and *P*=0.006) as shown in Table 2.

### 3.3. Comparative Analysis of the Changes in Ultrasound Parameters between the NMHR Group and the MHR Group

Table 3 shows that in the MHR group, the diameter changes reaching PR + CR, regular morphological changes, narrowing/disappearance of hyperechoic halos, clear borders, posterior echo changes, decreased blood flow grade, and decreased RI were higher than those of the NMHR group (all *P* < 0.05) as shown in Table 3.

### 3.4. Multivariate Analysis of the Degree of Pathological Remission

The results of single-factor analysis of *P* < 0.05 variables as a multifactor analysis of the independent variable have a diameter change, shape change rules, strong echo halo narrow/disappear, boundary clear, rear echo change, blood flow and RI level lower eight indicators, binary classification multivariable logistic regression analysis, screening method of the independent variables selection method step by step forward. The results show that ER, narrowing/disappearance of strong echo halo, Adler classification after NAC, PR + CR, and morphological change rule enter the model. The response rate of ER positive was 0.176 times that of negative (OR = 0.176, 95%CI: 0.046∼0.663). The remission rate of hyperechoic halo narrowing/disappearance was 10.661 times that of no narrowing/disappearing echogenic halo (OR = 10.661, 95% CI: 2.608∼43.568). Each time Adler increased by one grade, and the remission rate became 0.129 times the original (OR = 0.129, 95% CI: 0.050∼0.333). The remission rate of PR + CR positive was 5.846 times that of negative (OR = 5.846, 95% CI: 1.077∼31.742). The remission rate of the morphological change rule was 6.223 times that of the rule (OR = 6.223, 95% CI: 1.696∼22.824) as shown in Table 4.

### 3.5. Establishment of the Nomogram Model

**Figure 2:**
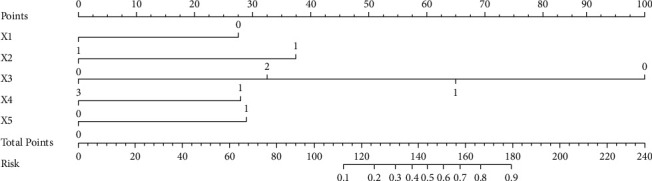
Nomogram predicting postoperative remission rate.

According to the risk factors screened out from the multivariate logistic regression analysis results, a nomogram model for predicting the degree of remission was established. After adding the specific scores of the five variable indicators, the total score is obtained, and the specific probability value of the patient's remission can be obtained by the corresponding probability line, as shown in Figure 2.

### 3.6. Evaluation of Nomograms

**Figure 3:**
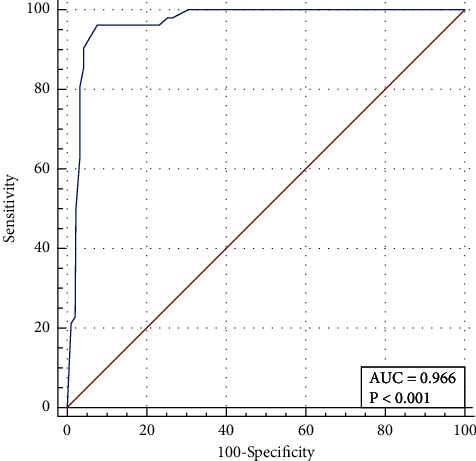
ROC analysis.

**Figure 4:**
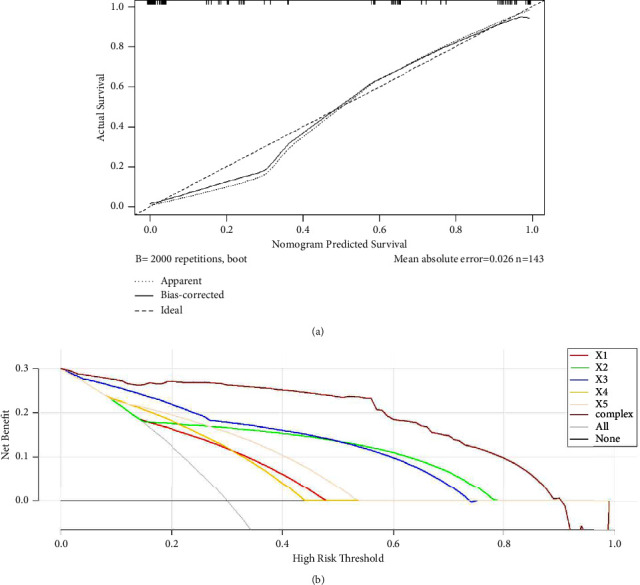
Calibration curve (a) and decision curve (b) of the nomogram model for predicting remission rate.

The area under the curve (AUC) was 0.996 (95%CI: 0.921–0.989), the sensitivity was 96.15%, and the specificity was 92.31%. PPV and NPV were 87.27% and 97.15%, respectively (Table 5 and Figure 3). The nomogram model was internally validated by Bootstrap self-sampling for 2000 times, and the resulting C-index for predicting remission rate was 0.966, indicating a good resolution (Figure 4(a)). The calibration curve results show that the average absolute error of coincidence between the predicted value and the real value is 0.026, and the predicted risk is close to the actual risk, indicating that the predicted coincidence is high. In the HRT range of approximately 0.0–0.9, the net benefit rate of the composite evaluation model was higher than that of the simple model (Figure 4(b)). The results of the Hosmer–Lemeshow test showed that = 8.430, *P*=0.393 > 0.05, indicating that through the HL test, there was no significant difference between the predicted value and the true value.

### 3.7. Typical Case Application

**Figure 5:**
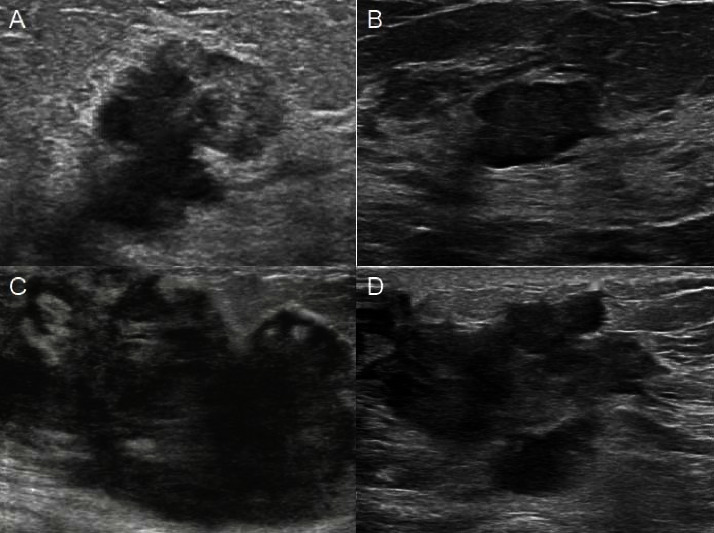
Ultrasound images of two patients who received NAC and belonged to the MHR group. (a, c) Images before NAC. (b, d) Images after NAC ends.

In the nomogram, by summing the scores of these 5 variables and locating them on a total subscale, the predicted probability of the degree of postoperative pathological response of the patient can be obtained. For example, Figures 5(a) and 5(b) are the ultrasound images of a 56-year-old female breast cancer patient before and after NAC, ER positive (0 points), the disappearance of hyperechoic halo after NAC (38 points), Alder grade 0 (100 points) points), the diameter change reached PR (28 points), and the morphological changes were more regular (30 points). The final total score is 196. The probability of predicting the degree of pathological remission as MHR was more than 90%, and the final MP grade was 5, which belonged to the MHR group.

Figures 5(c) and 5(d) are the ultrasound images of a 63-year-old female breast cancer patient before and after NAC, ER negative (28 points), hyperechoic halo narrowing after NAC (38 points), Alder grade 1 (66 points) points), the diameter change reached PR (28 points), and the morphological changes were not obvious (0 points). The final total score is 160. Judging from the experience of two senior physicians, this patient has a low probability of achieving MHR, but the model shows that the probability of predicting MHR is more than 70%, and the final MP grade is 4, which belongs to the MHR group. This shows that the model has a good predictive ability.

## 4. Discussion

Imaging examinations can not only evaluate the efficacy, pathological status, and prognosis of NAC but also help to choose the most appropriate surgical approach. Current research generally believes that magnetic resonance imaging (MRI) is more objective than ultrasound (Ultrasound, US). The degree of remission and prognosis has advantages, but due to the high cost, it has not been fully popularized in all parts of the world, especially in developing countries [16–19]. At the same time, some studies have shown that breast cancer patients with negative ER before NAC and high expression of Ki67 are more sensitive to chemotherapy and benefit more after chemotherapy, and the two may be sensitive factors for predicting the efficacy of chemotherapy [24, 25]. Some studies have shown that many changes will occur in the ultrasound images of patients who achieve pcR after NAC, such as clinical efficacy assessment achieves PR and CR, posterior echo attenuation disappears, internal echo increases, and the hyperechoic halo around the tumor narrows or disappears, and these are the relevant indicators of tumor NAC effective [26]. Different breast cancer patients have different sensitivities to neoadjuvant chemotherapy, and ultrasound diagnosis is an important means to assess the efficacy of neoadjuvant chemotherapy early and formulate individualized treatment plans for patients. Many studies have shown that the nomogram can be considered as an effective tool for predicting the degree of pathological remission after NAC [27, 28]. However, few studies have developed models for predicting NAC efficacy based on ultrasound and clinical indicators. In order to develop a new method that is clinically applicable, cost-effective, and easy to promote, this study combined commonly used two-dimensional gray-scale ultrasound and color Doppler ultrasound with immunohistochemical and serum markers commonly detected in clinics.

In this study, not only the ultrasound images before and after NAC were included but also the changes in parameters between them were analyzed. We believe that the changes in ultrasound parameters during treatment can more directly reflect the degree of pathological remission of NAC. Therefore, the pretreatment, posttreatment, changes in ultrasound image parameters, and commonly used clinical indicators are all incorporated into the nomogram-based prediction model for the degree of postoperative pathological remission after NAC, and these indicators are easy to obtain in daily clinical work. This means that ER(−), hyperechoic halo narrowing/disappearance, post-NAC Adler grade, PR + CR, and morphological changes are more likely to achieve significant responses. Based on the risk factors (ER, narrowing/disappearance of strong echo halo, Adler classification after NAC, PR + CR, and morphological change rule) selected from the results of multifactor logistic regression analysis, a line chart model was established to predict the remission degree. Evaluation of the model found that the nomogram performed well, and the AUC/C index under the ROC curve reached a respectable 0.96. Sensitivity and specificity were 96.15% and 92.31%, both satisfactory. PPV and NPV were 87.27% and 97.15%, respectively. This means that the nomogram performed well. It is helpful to clarify the independent risk factors of postoperative pathological remission and to provide guidance for the choice of subsequent treatment. The predicted values obtained by the nomogram are in good agreement with the actual observed values. The results showed that the mean absolute error of the agreement between the predicted value and the true value was 0.026, and the predicted risk was close to the actual risk, indicating that the degree of agreement for predicting postoperative pathological remission was high.

The emerging deep learning representation of ultrasound image features, based on pre-NAC and post-NAC ultrasound images, uses deep learning radiomics to establish a pCR prediction model, which can provide an effective diagnostic reference for clinical routine pCR identification [29, 30]. Studies have shown that during the whole process of preoperative NAC treatment, ultrasound can dynamically observe the changes in the tumor and evaluate the effectiveness of NAC, so that the treatment plan can be changed in time when it is ineffective [31]. In the nomogram established by some scholars, it was also found that diameter reduction after NAC was an important dependent factor for predicting pCR, and the characteristics of ultrasound images and the changes between these characteristics were related to pCR [32, 33]. The change in diameter after the end of NAC, i.e., whether PR and CR are achieved, is the most important feature and also occupies an important position in the nomogram constructed in our study.

Many studies have found that the Adler grade, RI, and PI of the lesions after effective chemotherapy are lower than those before chemotherapy [34]. There are also studies showing that CR is a valid and valuable surrogate prognostic factor for survival after treatment [35]. The results of this study show that the blood flow grade after NAC is an independent influencing factor of MHR. Previous studies have shown that two-dimensional gray-scale ultrasound features, including hyperechoic halos, and tumor morphology are closely related to the diagnosis of breast cancer. The disappearance of the hyperechoic halo in the nomogram and the changes in tumor morphology were considered to be associated with a significant degree of pathological remission.

## 5. Limitations and Prospects

### 5.1. Limitations

1. Since this is a single-center retrospective study, the exact parameters regarding the machine setup were not initially available. Furthermore, even for the same type of machine, the settings of different institutions may differ to some extent; therefore, it is difficult to assess whether the type of machine affects the parameters of the image and the performance of the prediction model.
2. There is a lack of prospective validation to determine the influence of sonographers on the future performance of the model.
3. The sonographer's judgment is subject to a certain degree. The evaluation of various characteristics of breast tumor ultrasonic images is qualitative and depends on the doctor's experience.
4. The sample size contained is insufficient, and the obtained results may be biased. Moreover, the follow-up time is short, which can be used as a reference for evaluating the short-term prognosis of breast cancer NAC, and multisample and long-term follow-up studies are still needed for long-term prognosis. Furthermore, additions and improvements are needed.

### 5.2. Prospects

1. This study developed a predictive model for the degree of pathological remission after NAC based on pre- and post-NAC ultrasound images and the changes in parameters between them and obtained good performance in an internal validation cohort. This model can provide an effective reference for evaluating the degree of pathological remission after routine surgery.
2. In the future, we hope to continue prospective studies of ultrasound using and comparing the effectiveness of this method in various molecular subtypes of breast cancer and larger sample sizes. At the same time, more factors were introduced into the nomogram, such as serum change percentage, lymph node metastasis status, and hope to predict the response before NAC administration, which will be the focus of our future research. This operation needs to be continuously accumulated and improved in practical applications, which will also be the direction of the next research work.
3. In the future, we hope to continue prospective studies with ultrasound to use and compare the effectiveness of this method in various molecular subtypes of breast cancer with larger sample sizes.

## 6. Conclusions

In conclusion, this study developed a predictive model for the degree of pathological remission after NAC based on US images before and after NAC, and the nomogram established by the combination of changes in ultrasound parameters and clinical indicators showed satisfactory efficiency, which means that the nomogram is a reliable method to predict the degree of postoperative pathological remission after NAC. It should be further explored in the future to give full play to the combination of changes in ultrasound image parameters and clinical indicators to better show the predictive value of US in predicting postoperative pathological remission [3].

## Figures and Tables

**Table 1:** Basic demographic data of patients.

Demographic indicators		NMHR (*n* = 93)	MHR (*n* = 54)	*P*
Age (years)		57.0 (49.0, 63.5)	52.5 (48.0, 57.0)	0.009
Whether menopause	No	49 (52.7)	33 (61.1)	0.322
	Yes	44 (47.3)	21 (38.9)	

History of birth and lactation	No	6 (6.5)	0 (0.0)	0.141
	Yes	87 (93.5)	54 (100.0)	

Family history	No	91 (97.8)	54 (100.0)	0.278
	Yes	2 (2.2)	0 (0.0)	

Lymph node metastasis	No	22 (23.9)	14 (25.9)	0.785
	Yes	70 (76.1)	40 (74.1)	

Period of treatment	<6 issues	39 (41.9)	12(22.2)	0.015
	≥6 periods	54 (58.1)	42 (77.8)	

Program	TAC	47 (50.5)	20 (37.0)	<0.001
	TCbHP	2 (2.2)	13 (24.1)	
	AT	11 (11.8)	8 (14.8)	
	TP	4 (4.3)	5 (9.3)	
	AC	8 (8.6)	1 (1.9)	
	TC	21 (22.6)	7 (13.0)	

Pathological type	Catheter	80 (86.0)	45 (83.3)	0.728
	Leaflet	6 (6.5)	3 (5.6)	
	Myeloid	4 (4.3)	2 (3.7)	
	Unknown	3 (3.2)	4 (7.4)	

ER	Feminine	32 (35.2)	39 (75.0)	<0.001
	Positive	59 (64.8)	13 (25.0)	

PR	Feminine	51 (56.0)	46 (88.5)	<0.001
	Positive	40 (44.0)	6 (11.5)	

CerbB-2	Feminine	7 (7.6)	3 (5.6)	0.893
	Positive	85 (92.4)	51 (94.4)	

Ki67	Feminine	20 (22.2)	3 (5.8)	0.010
	Positive	70 (77.8)	49 (94.2)	

**Table 2:** Comparative analysis of differences in ultrasound and serum indexes between the NMHR group and MHR group.

Index	Before therapy				After treatment		
	NMHR (*n* = 93)		MHR (*n* = 54)	*P*	NMHR (*n* = 93)	MHR (*n* = 54)	*P*
Form	Rule	3 (3.2)	1 (1.9)	0.622	5 (5.4)	3 (5.8)	1.000
	Irregular	90 (96.8)	53 (98.1)		88 (94.6)	49 (94.2)	

Direction	Level	83 (89.2)	51 (94.4)	0.442	85 (91.4)	46 (92.0)	1.000
	Vertical bit	10 (10.8)	3 (5.6)		8 (8.6)	4 (8.0)	

Strong echo halo	None	59 (63.4)	18 (33.3)	<0.001	66 (71.0)	52 (96.3)	<0.001
	Have	34 (36.6)	36 (66.7)		27 (29.0)	2 (3.7)	

Calcification	None	43 (46.2)	23 (42.6)	0.668	22 (23.7)	16 (32.0)	0.281
	Have	50 (53.8)	31 (57.4)		71 (76.3)	34 (68.0)	

Boundary	Clear	53 (57.0)	22 (40.7)	0.057	22 (23.7)	7 (13.0)	0.116
	Not clear	40 (43.0)	32 (59.3)		71 (76.3)	47 (87.0)	

Echo	Very low	16 (17.2)	5 (9.4)	0.412			0.662
	Low	74 (79.6)	44 (83.0)		69 (75.0)	35 (70.0)	
	Mix	2 (2.2)	3 (5.7)		3 (3.3)	1 (2.0)	
	Wait for an echo	1 (1.1)	1 (1.9)		20 (21.7)	14 (28.0)	

Rear echo attenuation	None	64 (68.8)	47 (87.0)	0.013	49 (52.7)	32 (59.3)	0.440
	Have	29 (31.2)	7 (13.0)		44 (47.3)	22 (40.7)	

Adler	Level 0	0 (0.0)	2 (3.7)	<0.001	10 (10.8)	38 (70.4)	<0.001
	Level 1	13 (14.0)	29 (53.7)		27 (29.0)	14 (25.9)	
	Level 2	46 (49.5)	22 (40.7)		38 (40.9)	2 (3.7)	
	Level 3	34 (36.6)	1 (1.9)		18 (19.4)	0 (0.0)	

Diameter	3.60 (2.40,5.05)		3.00 (2.08,4.80)	0.164	2.30 (1.40,4.00)	1.00 (0.70,1.80)	<0.001
RI	0.74 (0.68,0.80)		0.62 (0.60,0.68)	<0.001	0.68 (0.61,0.76)	0.00 (0.00,0.51)	<0.001
CEA	2.71 (1.68,5.71)		2.34 (1.41,5.18)	0.436	2.60 (1.60,3.81)	1.85 (1.28,3.07)	0.003
CA125	16.23 (10.74,25.22)		14.11 (10.29,24.74)	0.674	14.41 (10.67,19.52)	13.97 (10.19,17.27)	0.210
CA153	18.49 (12.22,28.50)		12.70 (7.95,21.83)	0.003	20.47 (15.73,28.55)	15.65 (12.48,23.61)	0.006
CA50	5.40 (2.61,11.00)		4.95 (2.70,9.65)	0.629	7.35 (4.30,11.25)	6.80 (3.90,11.20)	0.591

**Table 3:** Comparative analysis of the changes in ultrasound parameters between the NMHR group and the MHR group.

Variables and their classification		NMHR (*n* = 93)	MHR (*n* = 54)	*P* value
PR + CR	None	48 (51.6)	6 (11.1)	<0.001
	Have	45 (48.4)	48 (88.9)	

Morphology rules	None	64 (68.8)	9 (16.7)	<0.001
	Have	29 (31.2)	45 (83.3)	

Change of direction	None	81 (87.1)	46 (85.2)	0.745
	Have	12 (12.9)	8 (14.8)	

Hyperechoic halo narrows/disappears	None	86 (92.5)	20 (37.0)	<0.001
	Have	7 (7.5)	34 (63.0)	

Increased number of calcifications	None	54 (58.1)	40 (74.1)	0.051
	Have	39 (41.9)	14 (25.9)	

Clear boundaries	None	65 (69.9)	29 (53.7)	0.049
	Have	28 (30.1)	25 (46.3)	

Echo becomes high	None	73 (78.5)	36 (66.7)	0.114
	Have	20 (21.5)	18 (33.3)	

Rear echo change	None	70 (75.3)	32 (59.3)	0.042
	Have	23 (24.7)	22 (40.7)	

Decreased blood flow	None	49 (52.7)	10 (18.5)	<0.001
	Have	44 (47.3)	44 (81.5)	

RI decreased	None	38 (40.9)	1 (1.9)	<0.001
	Have	55 (59.1)	53 (98.1)	

**Table 4:** Multivariate analysis of the degree of pathological remission.

Variable	Multiactor		
	OR	95% CI	*P*
ER (control: negative)	0.176	0.046∼0.663	0.010
Hyperechoic halo narrows/disappears (control: negative)	10.661	2.608∼43.568	0.001
NAC after Adler (control: grade 0)	0.129	0.050∼0.333	<0.001
PR + CR (control: negative)	5.846	1.077∼31.742	0.041
Morphology rules (control: negative)	6.223	1.696∼22.824	0.006

**Table 5:** ROC analysis of emission degree and prediction probability.

Variable	AUC	Standard error	*P* value	95% CI	PPV (%)	NPV (%)
Prediction probability	0.996	0.059	<0.001	0.921∼0.989	87.72	97.15

## Data Availability

The data used to support the findings of this study are available from the authors upon request.
